# Polarization of macrophages in the tumor microenvironment is influenced by EGFR signaling within colon cancer cells

**DOI:** 10.18632/oncotarget.12207

**Published:** 2016-09-23

**Authors:** Weina Zhang, Lechuang Chen, Kai Ma, Yahui Zhao, Xianghe Liu, Yu Wang, Mei Liu, Shufang Liang, Hongxia Zhu, Ningzhi Xu

**Affiliations:** ^1^ Laboratory of Cell and Molecular Biology & State Key Laboratory of Molecular Oncology, Cancer Hospital, Chinese Academy of Medical Sciences and Peking Union Medical College, Beijing 100021, P.R. China; ^2^ State Key Laboratory of Biotherapy and Cancer Center, West China Hospital, Sichuan University, and Collaborative Innovation Center for Biotherapy, Chengdu 610041, P.R. China

**Keywords:** TAM, colon cancer, tumor microenvironment, EGFR, IGF-1

## Abstract

Epidermal growth factor receptor (EGFR) is a target of colon cancer therapy, but the effects of this therapy on the tumor microenvironment remain poorly understood. Our *in vivo* studies showed that cetuximab, an anti-EGFR monoclonal antibody, effectively inhibited AOM/DSS-induced, colitis-associated tumorigenesis, downregulated M2-related markers, and decreased F4/80^+^/CD206^+^ macrophage populations. Treatment with conditioned medium of colon cancer cells increased macrophage expression of the M2-related markers arginase-1 (Arg1), CCL17, CCL22, IL-10 and IL-4. By contrast, conditioned medium of EGFR knockout colon cancer cells inhibited expression of these M2-related markers and induced macrophage expression of the M1-related markers inducible nitric oxide synthase (iNOS), IL-12, TNF-α and CCR7. EGFR knockout in colon cancer cells inhibited macrophage-induced promotion of xenograft tumor growth. Moreover, colon cancer-derived insulin-like growth factor-1 (IGF-1) increased Arg1 expression, and treatment with the IGF1R inhibitor AG1024 inhibited that increase. These results suggest that inhibition of EGFR signaling in colon cancer cells modulates cytokine secretion (e.g. IGF-1) and prevents M1-to-M2 macrophage polarization, thereby inhibiting cancer cell growth.

## INTRODUCTION

Colorectal cancer (CRC) is the fifth and fourth most commonly diagnosed cancer in males and females, respectively, and is the fifth most common cause of cancer-related death in China [1]. Colitis associated cancer (CAC) is a type of CRC that results from inflammatory bowel disease (IBD) [2, 3]. Traditional colorectal cancer therapies include surgical resection, chemotherapy, and radiotherapy [4]. In recent years, studies have investigated the influence of the tumor microenvironment on carcinogenesis and therapeutic efficacies [5, 6]. Tumor-associated macrophages (TAMs) that infiltrate tumors are abundant in the tumor microenvironment. TAMs play important roles in chronic inflammation and in the tumor microenvironment and not only promote tumor cell growth, but also affect the efficacy of different antineoplastic therapies [7–10].

Macrophages are generally classified as the classical ‘M1’ type activated by Th1 cytokines or the alternative ‘M2’ type activated by Th2 cytokines [11–14]. M1-like macrophages play a central role in killing invading pathogens and tumor cells, secrete pro-inflammatory cytokines such as IL-12, TNF-a, CXCL10, IFN-γ, and exhibit high nitric oxide synthase (iNOS) expression. In contrast, M2-like macrophages are important in tissue repair and tumor progression, secrete anti-inflammatory cytokines such as IL-10, IL-13, and IL-4, and exhibit high mannose receptor (MR, CD206) and Arg1 expression [13, 15, 16]. TAMs typically display the M2-like phenotype [17, 18].

The EGFR signaling pathway plays a crucial role in colon cancer survival, growth, and metastasis [19–21]. EGFR is overexpressed in 60-80% of colon cancers, and clinical studies of EGFR-targeted drugs have already been conducted [22]. Moreover, EGFR-targeted therapy might not only directly inhibit tumor cell growth, but also modulate the tumor microenvironment. For example, the EGF/EGFR pathway modulates cytokine profiles in breast cancer. Cannabidiol inhibits the EGF/EGFR pathway and alters cytokine production in tumor cells, leading to a reduction in the numbers of total and M2 macrophages at the primary and secondary tumor sites [23]. Inhibition of EGFR signals in human colon cancer also influences cytokine secretion [24]. However, whether the EGFR signaling pathway influences macrophage polarization in colorectal tumor microenvironment has not yet been investigated.

In this study, we found that the EGFR pathway modulated macrophage numbers and polarization in colon cancer, and consequently influenced tumor growth. Furthermore, IGF-1 secreted from colon cancer cells also influenced macrophage polarization.

## RESULTS

### Cetuximab modulates macrophage polarization in an AOM/DSS mouse model

**Figure 1:**
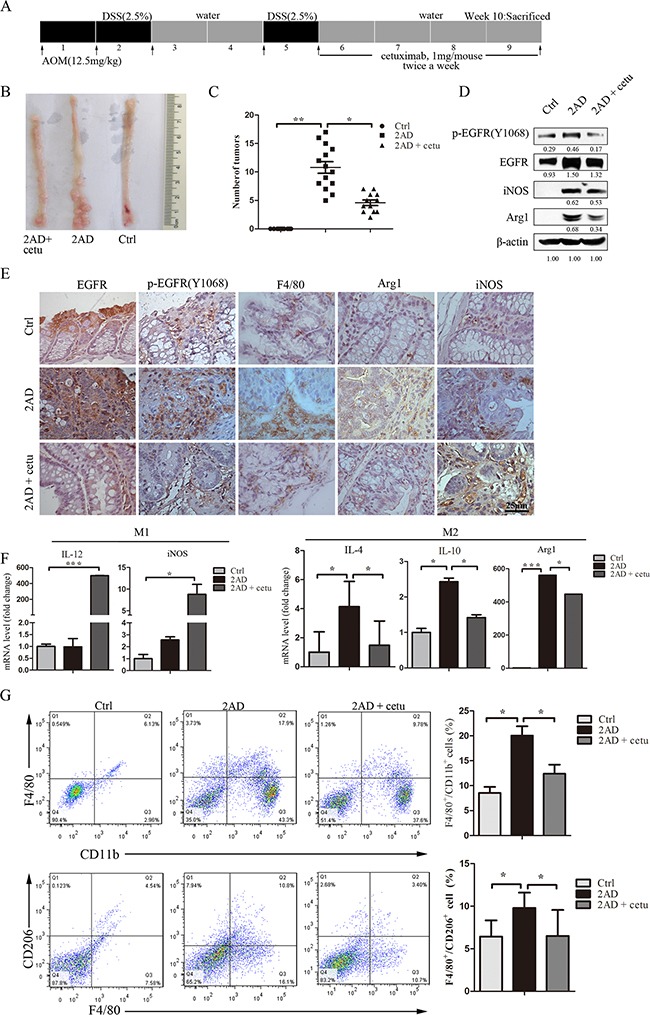
Cetuximab modulates macrophage polarization in an AOM/DSS mouse model **A.** Establishment of the AOM/DSS mouse model. AOM was injected intraperitoneally at 12.5 mg/kg body weight. After one week, mice were given drinking water containing 2.5% DSS for 5 days followed by 16 days of regular drinking water. After two cycles of DSS treatment, cetuximab (1 mg/mouse, twice a week) was injected intraperitoneally for a month, and the mice were then sacrificed. **B.** Representative images of colon tumors in normal (right), AOM/DSS (2AD) (middle), and cetuximab-treated AOM/DSS mice (2AD+cetu) (left). **C.** Tumor quantification. Cetuximab treatment (2AD + cetu) reduced tumor numbers compared to 2AD mice. **D.** p-EGFR (Y1068), EGFR, Arg1, and iNOS protein levels in normal mice, 2AD and 2AD+cetu mouse tumors were detected by Western blot. All experiments were repeated three times. **E.** Representative photomicrographs of immunostaining for p-EGFR (Y1068), PCNA, F4/80, Arg1, and iNOS. Scale bars: 25 μm. **F.** M1 marker (IL-12, iNOS) and M2 marker (IL-4, IL-10, Arg1) mRNA levels in normal mouse colon tissues, 2AD and 2AD+cetu mouse tumor tissues were evaluated by q-PCR. **G.** Percentages of CD11b^+^/F4/80^+^ and F4/80^+^/CD206^+^ cells in normal, 2AD, and 2AD+cetu mice colon tissues were detected by flow cytometry. Colon tissues were cut into small pieces (1-2 mm) and incubated with collagenase D (1- mg/mL), dispase II (1 mg/mL), and DNase I (100 μg/mL) for 30-45 min in a shaking incubator at 37°C, and single-cell suspensions were then incubated with antibodies. Bars represent means ± SD (n = 3) for each treatment. *p < 0.05; **p < 0.01; ***p < 0.001.

The AOM/DSS mouse model was created by intraperitoneal injection of the pro-carcinogen AOM followed by two cycles of DSS (2AD) exposure (Figure 1A). As expected, colonic tumors were visible in all AOM/DSS mice (Figure 1B). After the second DSS cycle, mice were treated for four weeks with saline or cetuximab (1 mg/mouse, twice a week), a dose previously shown to prevent tumor formation [25]. Notably, cetuximab reduced the number of tumors, and most tumors were smaller (< 2 mm), in cetuximab-treated 2AD mice (2AD + cetu) compared to saline-treated 2AD (2AD) mice (Figure 1B and 1C). Histopathologically, 2AD mice developed adenoma tumors with high-grade dysplasia, while 2AD+cetu mice developed low-grade dysplasia (Supplementary Figure S1A). PCNA staining was stronger in 2AD mice than in normal mice and 2AD + cetu mice (Supplementary Figure S1B). These results suggest that cetuximab effectively protected against AOM/DSS-induced, colitis-associated tumorigenesis.

Western blot results showed that levels of p-EGFR (Y1068), EGFR, Arg1, and iNOS proteins were higher in AOM/DSS mice than in normal mice. Treatment with cetuximab reduced levels of all of these proteins, except for iNOS, compared to 2AD mice (Figure 1D). Immunohistochemistry results were consistent with these findings. p-EGFR (Y1068) and EGFR levels were higher in 2AD mouse adenomas. F4/80-positive macrophage infiltration was present in 2AD and 2AD + cetu mice. Arg1 positive macrophages were abundant in 2AD mice, but rarely detected in normal mice and 2AD + cetu mice (Figure 1E). We then measured the expression of typical M1 and M2 macrophage marker mRNAs. Expression of iNOS and IL-12, which are typical M1 markers, did not differ between 2AD and normal mice, but were higher in 2AD + cetu mice (Figure 1F). In contrast, Arg1, IL-10, and IL-4, which are typical M2 markers, were higher in 2AD than in normal mice, and cetuximab treatment inhibited Arg1, IL-10, and IL-4 mRNA expression (Figure 1F).

Next, we analyzed macrophage populations in primary tumors using flow cytometry. 2AD mice had more total macrophages (F4/80^+^/CD11b^+^) and a higher percentage of M2 macrophages (F4/80^+^/CD206^+^) than normal mice, and cetuximab decreased both macrophage populations (Figure 1G). Taken together, these results suggest that cetuximab inhibits macrophage accumulation and M2 polarization in the AOM/DSS mouse model.

### Inhibition of the EGFR signaling pathway in colon cancer cells reduces M2-like macrophage polarization

**Figure 2:**
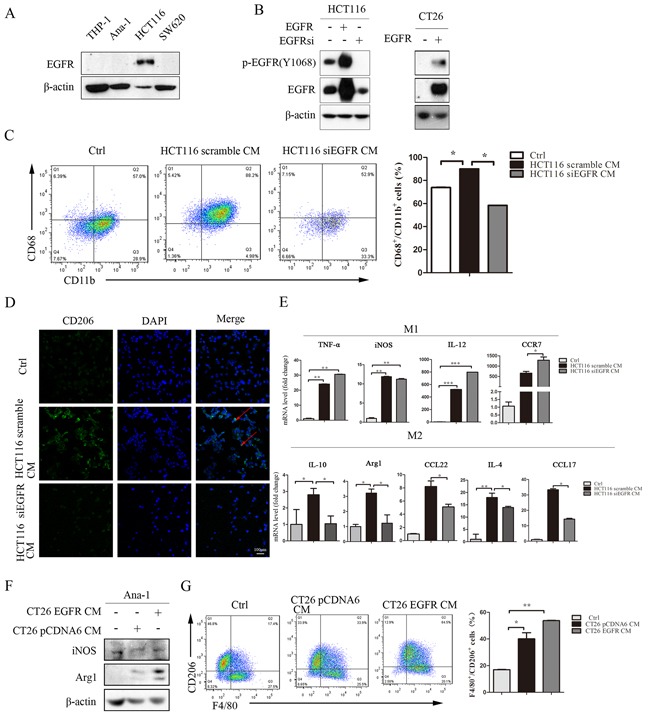
Inhibition of the EGFR signaling pathway in colon cancer cells prevents conditioned medium-induced M2-like macrophage polarization **A.** EGFR protein levels in THP-1, Ana-1, HCT116, and SW620 cells were detected by Western blot. **B.** HCT116 cells were cultured to 50% confluence and then transfected with human scramble siRNA, pCDNA6-EGFR WT plasmid, or EGFR siRNA. CT26 cells were cultured to 50% confluence and then transfected with human pCDNA6 vector or pCDNA6-EGFR WT plasmid for 48 h; the cells were then harvested for Western blots for EFGR. **C.** Percentages of CD68^+^/CD11b^+^ in THP-1 cells after 48 h of treatment with normal RPMI1640, HC116 scramble CM, or HCT116 siEGFR CM were detected by flow-cytometry. **D.** Immunofluorescent staining for CD206^+^ was measured in THP-1 cells after incubation with normal RPMI1640, HCT116 scramble CM, or HCT116 siEGFR CM. **E.** M1-related marker (TNF-α, iNOS, IL-12 and CCR7) and M2-related marker (IL-4, CCL17, CCL22, IL-10 and Arg1) mRNA levels were detected by q-PCR in THP-1 cells after incubation with normal RPMI1640, HCT116 scramble CM, or HCT116 siEGFR CM. Scale bars: 100 μm. **F.** Arg1 and iNOS protein levels in Ana-1 cells were detected by Western blot after incubation with CT26 pCDNA6 CM or CT26 EGFR CM. **G.** Percentages of F4/80^+^/CD206^+^ in Ana-1 cells after incubation with CT26 pCDNA6 CM or CT26 EGFR CM were detected by flow cytometry. Red arrows indicate CD206 expression in the cell membrane. Nuclei were counterstained with DAPI. Bars represent means ± SD (n = 3) for each treatment.*p < 0.05; **p < 0.01; ***p < 0.001.

A previous study found that macrophages express EGFR [26], but we did not detect EGFR protein expression in macrophages (Figure 2A), and cetuximab alone had no effect on macrophage polarization (Supplementary Figure S2). It is possible that the EGFR monoclonal antibody cetuximab does not directly influence macrophage polarization in the AOM/DSS mouse model. Cetuximab might inhibit EGFR signaling in colon cancer cells and alter the secretion of other factors into the tumor microenvironment, consequently preventing macrophage polarization. To investigate this possibility, we overexpressed EGFR in HCT116 and CT26 cells, and knocked down EGFR expression in HCT116 cells (Figure 2B). Cancer cell conditioned media (CM) were then harvested and used to treat macrophage cells. CM from HCT116 cells induced the polarization of THP-1 cells into CD68^+^/CD11b^+^ macrophages (Figure 2C) and CD206-positive macrophages (Figure 2D). In addition, the expression of M1 and M2 macrophage marker mRNAs increased in HCT116 CM-treated THP-1 cells. In HCT116 siEGFR CM-treated THP-1 cells, M2-related markers IL-10, Arg1, CCL17, CCL22, and IL-4 were downregulated, but M1-related markers IL-12, CCR7, and TNF-α were upregulated, compared to HCT116-CM treated THP-1 cells (Figure 2E).

Next, we incubated Ana-1 cells with CT26 or CT26 EGFR CM. Arg1 protein level and F4/80^+^/CD206^+^ positive cells increased after treatment with CT26 EGFR CM, while iNOS protein levels did not change after either CT26 CM or CT26 EGFR CM treatment (Figure 2F and 2G).

We also treated Ana-1 and bone marrow-derived macrophage (BMDM) cells with media conditioned by HCT116, SW480, and SW620 colon cancer cells and obtained similar results (Supplementary Figure S3-S5). Taken together, these results suggest that EGFR signaling activity in colon cancer cells might promote the polarization of M1-like macrophages into M2-like macrophages.

### EGFR knockout in colon cancer cells inhibits macrophage-induced xenograft tumor growth

**Figure 3:**
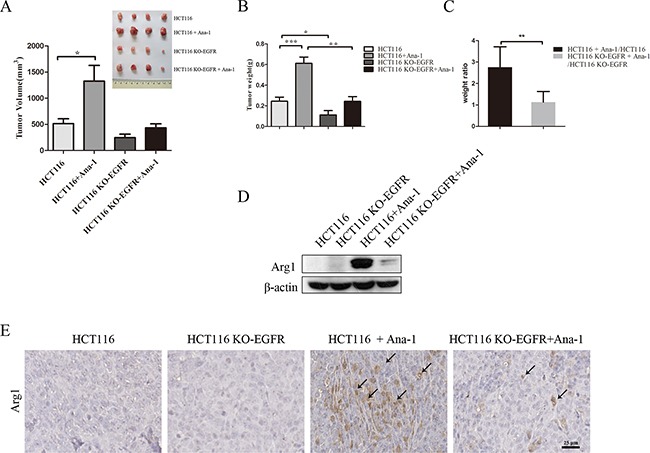
EGFR knockout in colon cancer cells inhibits macrophage-induced promotion of xenograft tumor growth **A.** Mouse tumor volume quantification (bottom left). Images (top right) of tumors resulting from subcutaneous injection of HCT116 cells alone, HCT116 plus Ana-1 cells, HCT116 KO-EGFR cells alone, and HCT116 KO-EGFR plus Ana-1 cells (n=4) into Balb/c nude mice. After 18 days, the mice were sacrificed and tumors were excised. The experiment was repeated twice. **B.** Mouse tumor weights were measured. **C.** Weight ratio of mice receiving HCT116 plus Ana-1/HCT116 injections compared to HCT116 KO-EGFR plus Ana-1/HCT116 KO-EGFR injections. **D.** Arg1 protein levels were detected by Western blot in HCT116, HCT116 KO-EGFR, HCT116 plus Ana-1, and HCT116 KO-EGFR plus Ana-1 mouse tumors. **E.** Immunostaining for Arg1 in xenograft mouse tumor tissues. Scale bars: 50 μm. Bars represent means ± SD (n = 3) for each treatment. *p < 0.05; **p < 0.01.

To investigate whether EGFR signaling activity in colon cancer cells stimulates Ana-1 macrophage polarization and how this polarization affects tumor growth, we subcutaneously injected HCT116 or HCT116 KO-EGFR cells with or without Ana-1 cells into Balb/c athymic nude mice. Injection of EGFR knockout HCT116 cells reduced tumor growth, and injection of Ana-1 cells with either HCT116 or HCT116 KO-EGFR cells increased tumor growth (Figure 3A, 3B). However, EGFR knockout in HCT116 cells dramatically reduced Ana-1-induced tumor growth; the tumor promotion efficiency of Ana-1 cells decreased from 2.7-fold with HCT116 cells to 1.3-fold with HCT116 KO-EGFR cells (Figure 3C).

Western blot showed that Arg1 protein was undetectable in HCT116 and HCT116 KO-EGFR tumors, but Arg1 levels increased in tumors induced by the injection of both HCT116 and Ana-1 cells compared to those induced by HCT116 KO-EGFR and Ana-1 cells (Figure 3D). IHC confirmed that HCT116 KO-EGFR plus Ana-1 tumors had fewer Arg1^+^ macrophages than HCT116 plus Ana-1 tumors (Figure 3E). HE staining of xenograft tissues is shown in Supplementary Figure S6A. Additionally, q-PCR showed that expression of the M1-related markers iNOS and CXCL10 was higher, while Arg1 expression was lower, in HCT116 KO-EGFR plus Ana-1 tumors than in HCT116 plus Ana-1 tumors (Supplementary Figure S6B). These results indicate that inhibition of the EGFR signaling pathway in HCT116 cells prevents M2 macrophage polarization and consequently inhibits tumor growth *in vivo*.

### Colon cancer-derived IGF-1 promotes M2-like macrophage polarization

**Figure 4:**
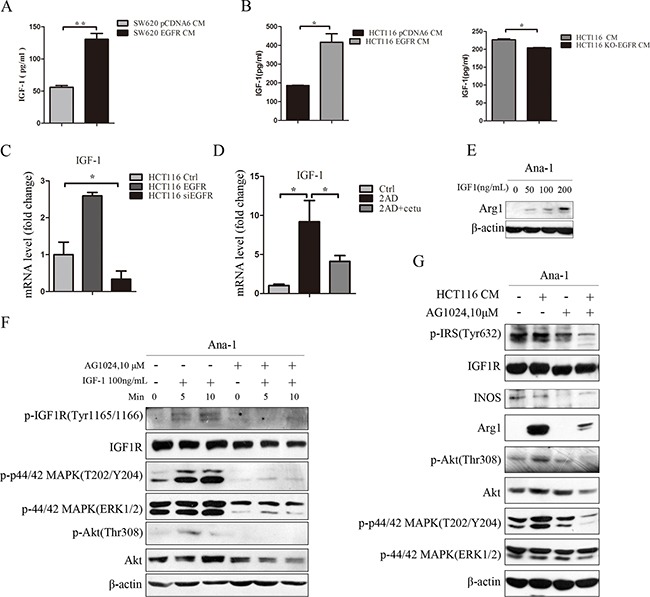
Colon cancer-derived IGF-1 promotes M2-like macrophage polarization **A.** IGF-1 concentrations were determined by ELISA in SW620 and SW620 EGFR cell culture media. SW620 cells were cultured to 50% confluence and then transfected with human pCDNA6 vector or pCDNA6-EGFR WT plasmid. After 24 h, cells were transferred to normal medium and cultured for an additional 24 h. Conditioned medium from SW620 cells was collected, centrifuged at 3000 rpm for 10 minutes, and stored at −80°C until use. **B.** IGF-1 concentrations in HCT116 and HCT116 EGFR (left) and HCT116 and HCT116 KO-EGFR (right) cell culture media were measured by ELISA. **C.** IGF-1 mRNA levels in HCT116, HCT116 EGFR, and HCT116 siEGFR cells were measured by q-PCR. **D.** IGF-1 mRNA levels in normal, 2AD, and 2AD+cetu mouse tissues were measured by q-PCR. **E.** Arg1 protein levels were detected by Western blot in Ana-1 macrophages after treatment with mouse recombinant IGF1 (0, 50, 100, or 200 ng/mL) for 48 h. **F.** Levels of IGF1R signaling pathway-related proteins were detected by Western blot. Ana-1 cells were harvested after treatment with 100 ng/mL mouse recombinant IGF-1 for 5 or 10 min, or after pretreatment with AG1024 for 30 min followed by 100 ng/mL mouse recombinant IGF-1 for 5 or 10 min. **G.** Protein levels were detected by Western blot in Ana-1 cells. Ana-1 cells were pretreated with AG1024 for 30 min, and medium was then replaced with fresh RPMI 1640 with AG1024 (10 mM) or HCT116 CM with AG1024 (10 mM) followed by an additional 48 h of culture. Cells were harvested and levels of Arg1, iNOS, and IGF1R signaling pathway-related proteins p-IRS(Tyr632), IGF1R, p-p44/42 MAPK(T202/Y204), p-44/42 MAPK(Erk1/2), p-Akt(Thr308), and Akt were measured by Western blot. Bars represent means ± SD (n = 3) for each treatment. *p < 0.05; **p < 0.01.

To investigate the mechanism by which conditioned medium promoted macrophage polarization, we performed a cytokine antibody array to compare differences between HCT116 siEGFR CM and HCT116 CM. We found that the IGF-1 concentration in HCT116 siEGFR CM was 53% of that in HCT116 CM (unpublished data). IGF-1 is a cytokine and a hormone factor, and might play a role in macrophage polarization [27]. Next, we examined whether IGF-1 levels in colon cancer CM were regulated by the EGFR pathway. Soluble IGF-1 in colon cancer CM was detected by ELISA. IGF-1 concentrations were higher in SW620 EGFR CM than in SW620 CM (Figure 4A). Overexpression of EGFR increased, and EGFR knockout decreased, IGF-1 secretion and mRNA expression in HCT116 cells (Figure 4B and 4C). We also investigated IGF-1 mRNA expression in AOM/DSS mouse colon tissues; IGF-1 expression was higher in 2AD mice than normal mice, and cetuximab treatment inhibited this increase in IGF-1 expression (Figure 4D).

To investigate the role of IGF-1 in macrophage polarization, we stimulated Ana-1 cells with recombinant IGF-1 (R&D) for 48 h. Western blot analysis showed that IGF-1 increased Arg1 protein levels in a dose-dependent manner (Figure 4E). IGF-1 also increased phosphorylation of the IGF1R, Akt, and MAPK signaling proteins (Figure 4F). Pretreatment with an Akt inhibitor abolished IGF-1-induced Arg1 expression in Ana-1 cells (Supplementary Figure S7A). Additionally, pretreatment with the IGF1R inhibitor AG1024 abolished IGF-1-induced IGF1R signaling pathway activation in Ana-1 cells (Figure 4F). HCT116 CM activated IGF1R signaling in Ana-1 cells and increased the expression of the M2 marker Arg1. After pretreatment with AG1024, HCT116 CM-induced IGF1R pathway activation was inhibited and Arg1 was downregulated in Ana-1 cells (Figure 4G). Human IGF-1 neutralizing antibody (R&D) also inhibited IGF-1 induced Arg1 expression (Supplementary Figure S7B). IGF-1 secretion was higher in EGF-stimulated HCT116 cells than in EGF-stimulated HCT116 KO-EGFR cells (Supplementary Figure S7C). Overall, these results demonstrate that IGF-1 from colon cancer cells induced M2 macrophage polarization, and inhibition of EGFR in HCT116 cells reduced both IGF-1 secretion and HCT116 CM-induced macrophage polarization.

## DISCUSSION

The EGFR signaling pathway plays a critical role in colonic tumorigenesis [28] and is a target of many cancer therapies. The anti-EGFR monoclonal antibodies cetuximab (Erbitux™, Bristol Myers Squibb and Merck KGaA) and panitumumab (VectibixTM, Amgen) are approved for the treatment of metastatic colorectal cancer (mCRC) [29, 30]. In recent years, EGFR-targeted therapy has been reported to not only inhibit tumor cells, but also reduce the secretion of GM-CSF, CCL3, TGF-α, bFGF, and VEGF, further affecting the tumor microenvironment [23, 24]. In this study, we found cetuximab reduced both tumor growth in an AOM/DSS mouse model and F4/80^+^/CD206^+^ macrophage populations. Experimental colitis colonic and peritoneal macrophages and human CD14^+^ monocytes have been reported to express EGFR [26, 31], but we did not detect EGFR expression in Ana-1 and THP-1 cells here (Figure 2A). Cetuximab might therefore modulate the secretion of various factors from tumor cells and thus inhibit macrophage accumulation and polarization.

To investigate this possibility, we incubated macrophages with medium conditioned by colon cancer cells and found that M2 polarization increased. EGFR overexpression promoted M2 polarization, whereas inhibition of the EGFR pathway prevented M2 polarization. Lewis lung carcinoma tumor conditioned medium increases the production of immunosuppressive factors in macrophages and decreases phagocytosis activity [32]. Melanoma conditioned-media is very effective for differentiating macrophages *in vitro* [33]. Additionally, signal molecules produced by tumor cells, such as lactate, HRG/PIGF, chemokine ligand 2 (CCL2), soluble colony-stimulating factor 1 (sCSF1), and POSTN, play critical roles in macrophage polarization [34–39]. Our results suggest that inhibition of the EGFR pathway might alter the components of conditioned medium to contribute to macrophage polarization. We confirmed this possibility by analyzing the secreted cytokine profiles of HCT116 cells after EGFR knockdown. EGFR knockout inhibited the secretion of IGF-1, which plays a key role in cell growth, differentiation, survival, transformation, and metastasis. IGF-1 is overexpressed in pancreatic, colon, breast, and ovarian cancers [40–43]. Our results also indicated that colon cancer tumor cells secrete IGF-1. Cancer associated fibroblasts [44] and TAMs [45] also secrete IGF-1 and promote tumor progression. Additionally, IGF-1 alters macrophage numbers and activity in liver microenvironments in obese mice [27].

We then investigated the role of IGF-1 in macrophage polarization. When the IGF-1 ligand binds to IGF1R, the immediate substrate insulin receptor substrate protein (IRS-1) is phosphorylated and recruits effectors containing SH2 or PTB domains to modulate the IGF1R pathway [46–48]. Here, IGF-1 directly promoted M2 macrophage polarization by activating the IGF1R signaling pathway, demonstrating for the first time that this pathway is important in macrophage polarization. Furthermore, we found that Akt signaling, which occurs downstream of IGF1R, was associated with macrophage polarization. Then we injected cancer cells and macrophages together to investigate the role of macrophage polarization in tumor growth. Ana-1 cells promoted tumor growth in the xenograft model, and most Ana-1 cells displayed the M2 phenotype. Moreover, EGFR knockout in HCT116 cells dramatically reduced the M2 macrophage population and tumor growth. Our results indicate that the tumor microenvironment induced M2 polarization in Ana-1 cells and that TAMs promote colon tumor growth. Inhibition of EGFR in HCT116 cells dramatically reduced TAM polarization and reduced tumor growth. Similar results have been reported for prostate cancer cells mixed with RAW264.7 cells. RAW264.7 cells promoted M2 polarization, which in turn promoted angiogenesis and tumor growth *in vivo* [36].

Colon cancer tissues contain large numbers of TAMs, which comprise the majority of immune cells within these tumors. Some studies have found that TAMs promote tumor progression in CRC patients; CD68^+^ macrophages are used as a marker of progression, and CD163^+^ macrophages are associated with early local recurrence and reduced survival times [49, 50]. Other studies indicate that macrophages inhibit tumor progression in CRC patients [51, 52]. However, the M1/M2 ratio in CRC patients is much higher than in prostate cancer patients, suggesting that M1 macrophages may be more important in CRC [52]. It is possible that the M1/M2 ratio rather than the total number of macrophages determines whether these cells promote or inhibit tumor growth. Moreover, TAMs that infiltrate the tumor invasive margin may be exposed to different tumor microenvironment signals than those that infiltrate the tumor stroma, possibly accounting for the different effects of TAMs on colon tumor growth [51, 53]. Because they are exposed to fewer signals produced by tumor cells, anti-tumor M1 polarization might predominate in peritumoral macrophages; in contrast, tumor microenvironment signals might increase pro-tumor M2 polarization in intratumoral macrophages. In our xenograft model, differences in the numbers of M2 macrophages rather than in total numbers of macrophages, which were similar among the groups, determined whether these cells inhibited or promoted tumor growth. Therefore, blocking tumor-induced M2 macrophage polarization might be a potential treatment strategy for inhibiting tumor growth.

**Figure 5:**
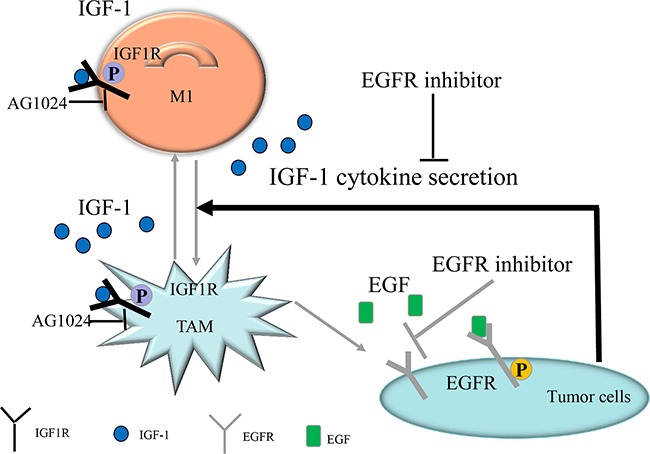
Diagrammatic model of EGFR pathway-modulated macrophage polarization in colon cancer IGF-1-induced IGF1R pathway activation might promote TAM polarization. EGFR inhibition in colon cancer cells altered the secreted cytokine profile. Secreted IGF-1 concentrations decreased when EGFR was inhibited in colon cancer cells, and IGF-1-induced TAM polarization was inhibited; experiments using the IGF1R inhibitor AG1024 and IGF-1 neutralizing antibody confirmed this effect. In conclusion, EGFR signaling pathway activity promoted IGF-1 secretion, which in turn induced TAM polarization.

In conclusion, our results suggest that inhibition of the EGFR signaling pathway in colon cancer cells alters cytokine secretion (e.g. IGF-1) and prevents M1- to M2-like polarization in macrophages, thus inhibiting cancer cell growth (Figure 5). Inhibiting such macrophage polarization might be a promising novel method for treating cancer.

## MATERIALS AND METHODS

### Cell lines

HCT116, SW480, THP-1, SW620, CT26, and Ana-1 cells were maintained in RPMI1640 (Bioroc™, China) supplemented with 10% heated-inactivated fetal bovine serum, 100 UI/mL penicillin, and 100 μg/mL streptomycin. Bone marrow derived macrophage (BMDM) cells were maintained in DMEM (Bioroc™, China) supplemented with 10% heated-inactivated fetal bovine serum, 100 UI/mL penicillin, and 100 μg/mL streptomycin. Cells were cultured in a humidified environment at 37°C with 5% CO_2._ Lipofectamine 3000 transfection reagent (Invitrogen, Carlsbad, CA, USA) was used for the transfection assay according to the manufacturer's instructions. EGFR small interfering RNA (siRNA) (NM_005228, sequence: 5′AGCUAUGAGAUGGAGGAAGACGGCG3′) and scrambled negative control siRNA were purchased from Integrated DNA Technologies (IDT, Coralville, IA, USA). PCDNA6-EGFR WT plasmid was provided by Mien-Chie Hung (Addgene plasmid # 42665) [54]. Target sequences for CRISPR interference were designed using this website: http://tools.genome-engineering.org. The human EFGR target sequences (sense: CTCTAAAACACTCGCCGGGCAGAGCGCAG, anti-sense: AAACACCGTACTACTAGACGGGGATGT) were cloned into the Cas9/gRNA (Puro-GFP) Vector (Viewsolid Biotech, China). After transfection and selection with puromycin, knockout clones (HCT116 KO-EGFR cells) were identified by Western blot. Genomic mutations were identified by genome sequencing.

### Isolation of bone marrow-derived macrophages (BMDMs)

BMDMs grown in macrophage colony-stimulating factor (M-CSF) were generated as previously described [35]. Briefly, BMDMs were isolated from the femurs of 4-5-week old C57 BL/6J mice, filtered through a 70 μm filter, and cultured in DMEM media supplemented with 10% heat-inactivated-FCS in the presence of M-CSF (10 ng/mL, R&D Systems). On day 3, non-adherent cells were discarded and adherent cells were cultured for an additional 3 days. On day 7, the culture medium was replaced with medium conditioned by tumor cells; 48 h later, the cells were collected and used in subsequent experiments.

### Establishment of the AOM/DSS mouse model and the tumor xenograft model

The establishment of the AOM/DSS mouse model was conducted as previously described [55]. Briefly, C57BL/6J mice (7 weeks old, approximate body weight 18-20 g) were injected intraperitoneally with azoxymethane (AOM) (12.5 mg/kg; Sigma-Aldrich, St. Louis, MO, USA). Seven days after injection, mice were treated with 2.5% Dextran sodium sulfate (DSS) (NW36, 000-50,000Da, MP Biomedicals, USA) in drinking water for 5 consecutive days, followed by 16 days of normal drinking water. This DSS treatment was repeated for one additional cycle. After the second cycle, cetuximab (Merck KGaA, Germany) (1 mg/mouse, twice a week) was injected for one month.

For the tumor xenograft model, 5-week-old female Balb/c athymic nude mice received 0.1 mL subcutaneous injections of 1×10^7^/mL HCT116 or HCT116KO-EGFR cells with or without 1×10^6^/mL Ana-1 cells. After 18 days, the mice were sacrificed. Tumor tissues were weighed and tumor volumes were calculated according to the formula length×width^2^×0.5. All experimental procedures using animals were reviewed and approved by the Institutional Animal Care and Use Committee (IACUC) at the Cancer Hospital of the Chinese Academy of Medical Science.

### Treatment of macrophages with colon cancer cell conditioned medium

Colon cancer cells were cultured to 50% confluence and transfected with human EGFR siRNA or pCDNA6A-EGFR WT plasmid for 24 h. After replacement with fresh medium, cells were cultured for an additional 24 h. Tumor cell conditioned medium was collected and centrifuged at 3000 rpm for 10 minutes. THP-1 cells were pretreated with 320 nM phorbol 12-myrisatate13-acetate (PMA, Sigma-Aldrich) for 72 h and thoroughly washed five times with 0.9% saline, and Ana-1 and THP-1 cells were then cultured with 2 mL conditioned medium for 48 h. Ana-1 cells were then incubated with human and mouse recombinant IGF-1 (R&D Systems, Minneapolis, MN, USA) for 48 h. IGF1R signaling was blocked with IGF1R inhibitor AG1024 (Selleck Chemicals, Texas, USA) and IGF-1 neutralizing antibody (R&D Systems) to confirm the role of IGF-1 in conditioned medium-driven macrophage polarization.

### Flow cytometry

Isolation of primary mouse macrophages from colon was performed as described previously [56]. Briefly, colon tissues were cut into small pieces (1-2 mm) and incubated in 10 mL PRMI 1640 medium with 10 mM HEPES and 5% fetal bovine serum containing 10 mg (1 mg/mL) collagenase D (Sigma-Aldrich), 10 mg (1 mg/mL) dispase II (Roche, Germany), and 100 μL 10 mg/ml DNase I (100 μg/mL) (Sigma-Aldrich) for 30-45 min in a shaking incubator at 37°C. For Ana-1 and BMDM cells, single-cell suspensions from mouse colon tissues were incubated with APC-anti-mouse CD11b and FITC-anti-mouse F4/80 (Affymetrix eBioscience, USA), or APC-anti-mouse F4/80 and FITC-anti-mouse CD206 (MMR) (Biolegend, San Diego, CA) antibodies. For THP-1 cells, single-cell suspensions were incubated with FITC anti-human CD68 intracellular marker before permeabilization with a BD Cytofix/Cytoooperm™ Fixation/Permeabilization Kit and incubation with PE-Cy™7 mouse anti-human CD11b/mac-1 (BD Biosciences, USA) antibody. Isotype control antibodies (Biolegend, eBioscience and BD Biosciences) were used as the negative control. All cells were incubated with antibodies for 30 min and washed with phosphate-buffered saline (PBS) before acquisition on a LSRII (BD, USA) and analysis with FlowJo software (Tree Stat, OR).

### Western blot

Whole cell lysates were prepared using a lysis buffer (Cell Signaling Technology, CST, Danvers, MA, USA) and protein concentration was determined using the Pierce™ BCA Protein Assay kit (Thermo Scientific). Western blots were performed as previously described [57]. The antibodies included β-actin (Sigma-Aldrich), p-IGF1R (Tyr1165/1166), Arginase1 (H-52), NOS2 (N-20), and p-IRS (Tyr632) (Santa Crus Biotech., Santa Cruz, CA, USA), and p-EGFR (Tyr1068), p-p44/42 MAPK (T204/Y202), EGFR, Akt, p44/42 MAPK (Erk1/2), IGF1R (CST), and p-Akt (Thr308) (Affinity Biosciences Inc.)

### Quantitative real-time polymerase chain reaction (q-PCR)

Total RNA was extracted with TRIZOL reagent (Invitrogen) according to the manufacturer's protocol. 0.5 μg of total RNA were used as the template for synthesizing cDNA with PrimeScript™ RT Master Mix (Perfect real time) (Takara, Dalian, China). q-PCR was performed on the Step-One Plus Real-Time PCR System (Applied Biosystems, Carlsbad, CA, USA) with Power SYBR Green PCR Master Mix (Applied Biosystems) and analyzed using StepOne Software. GAPDH was used as an internal control. Sequences of the PCR primers [58–62] are showed in Table 1.

**Table 1:** Primers used for q-PCR

Species	Oligo name	Sense	Anti-sense
human	GAPDH	GTGAAGGTCGGAGTCAACGG	CTCCTGGAAGATGGTGATGGG
	IL-10	TCCCTGTCAAAACAAGAGCA	ATAGAGTCGCCACCCTGATG
	IL-4	CTGTGCTCCGGCAGTTCTA	ACGTACTCTGGTTGGCTTCC
	IL-12	GCGGAGCTGCTACACTCTCT	GGTGGGTCAGGTTTGATGAT
	TNF-α	TGTAGCAAACCCTCAAGCTG	TTGATGGCAGAGAGGAGGTT
	iNOS	TCCAAGGTATCCTGGAGCGA	CAGGGACGGGAACTCCTCTA
	Arg1	ACGGAAGAATCAGCCTGGTG	GTCCACGTCTCTCAAGCCAA
	IGF-1	TGGATGCTCTTCAGTTCGTG	TGGTAGATGGGGGCTGATAC
	CCL17	ACTTCAAGGGAGCCATTCCC	CCTGCCCTGCACAGTTACAA
	CCL22	ATCGCCTACAGACTGCACTC	GACGGTAACGGACGTAATCAC
	CCR7	TGTACGAGTCGGTGTGCTTC	GGTAGGTATCCGTCATGGTCTTG
mouse	GAPDH	AGGTCGGTGTGAACGGATTTG	TGTAGACCATGTAGTTGAGGTCA
	INOS	GTTCTCAGCCCAACAATACAAGA	GTGGACGGGTCGATGTCAC
	CXCL10	CCAAGTGCTGCCGTCATTTTC	GGCTCGCAGGGATGATTTCAA
	Arg1	TGGCTTGCGAGACGTAGAC	GCTCAGGTGAATCGGCCTTTT
	IL-4	GGTCTCAACCCCCAGCTAGT	GCCGATGATCTCTCTCAAGTGAT
	IL-10	GCTCTTACTGACTGGCATGAG	CGCAGCTCTAGGAGCATGTG
	IL-12	ACTCTGCGCCAGAAACCTC	CACCCTGTTGATGGTCACGAC
	IL-13	GGATATTGCATGGCCTCTGTAAC	AACAGTTGCTTTGTGTAGCTGA
	IGF-1	CTGGACCAGAGACCCTTTGC	GGTGCCCTCCGAATGCT
	CCL17	AGTGCTGCCTGGATTACTTCAAAG	CTGGACAGTCAGAAACACGATGG
	CCL22	TAACATCATGGCTACCCTGCG	TGTCTTCCACATTGGCACCA
	CCR7	TGAGGTCACGGACGATTACAT	GTAGGCCCACGAAACAAATGAT

### Immunohistochemistry and Immunofluorescence

Sections (5 μm thick) of paraffin-embedded tissue were placed on glass slides, rehydrated, incubated with 3% hydrogen peroxide to quench endogenous peroxidase activity, and then blocked by incubation with 5% bovine serum albumin in PBS. Sections were then incubated with the arginase1 (N-20, Santa Cruz Biotech), PCNA (FL-261, Santa Cruz Biotech), F4/80 (MCA497GA, AbD Serotec), iNOS (Abcam, Cambridge, UK), EGFR, and p-EGFR (Y1068, CST) antibodies. Immunofluorescence was performed as previously described [63]. THP-1 cells were incubated with anti-mannose receptor antibody (Abcam).

### ELISA analysis

Cytokine IGF-1 levels were measured by enzyme-linked immunosorbent assay (ELISA) using an ELISA kit (R&D Systems). The ELISA was performed according to the manufacturer's protocol.

### Statistical analysis

All results are expressed as means ± SD. Paired, two-tailed Student's *t-*tests were used to identify significant differences between treatment and control groups. A *p* value of less than 0.05 was considered statistically significant. All analyses were conducted with GraphPad Prism5 software. Figures show the averages of three independent experiments.

## SUPPLEMENTARY FIGURES


