# Supplementary material for: Polarization of macrophages in the tumor microenvironment is influenced by EGFR signaling within colon cancer cells
Source: Oncotarget. 2016 Sep 23;7(46):75366–78. doi: 10.18632/oncotarget.12207 (PMC5342747; doi:10.18632/oncotarget.12207)
Supplement: Supplementary file 1 [file oncotarget-07-75366-s001.pdf]

Polarization of macrophages in the tumor microenvironment is influenced by EGFR signaling within colon cancer cells

SUPPLEMENTARY FIGURES

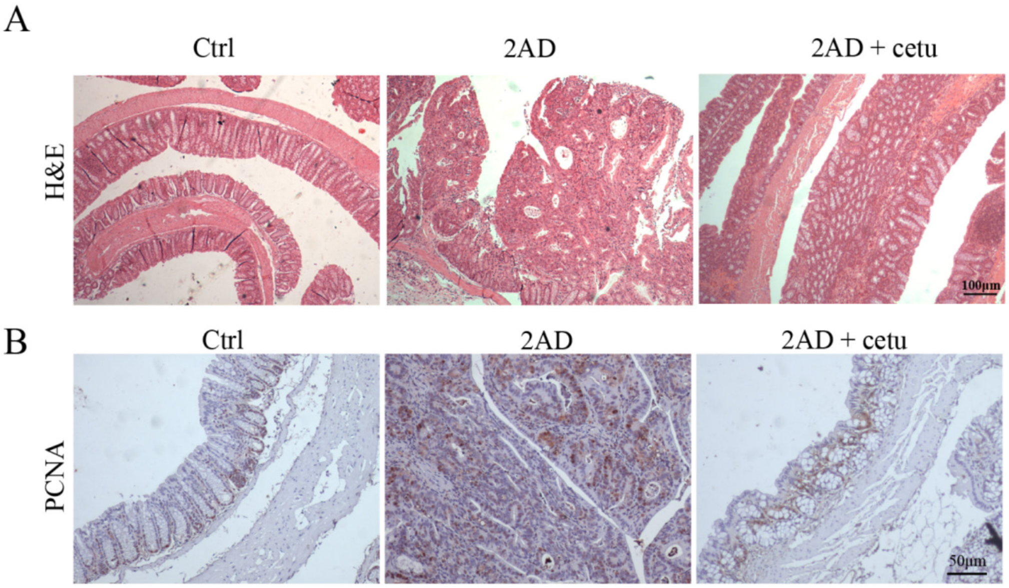

Supplementary Figure S1: Establishment of AOM/DSS mouse model. A. Histology of colon tissues in normal mice (Ctrl), AOM/DSS (2AD) and cetuximab treated 2AD (2AD + cetu) mice. Scale bars: 100 µm. **B.** Representative photomicrographs of immunostaining for PCNA in normal mice (Ctrl), 2AD mice and 2AD + cetu mice. Scale bars: 50 µm.

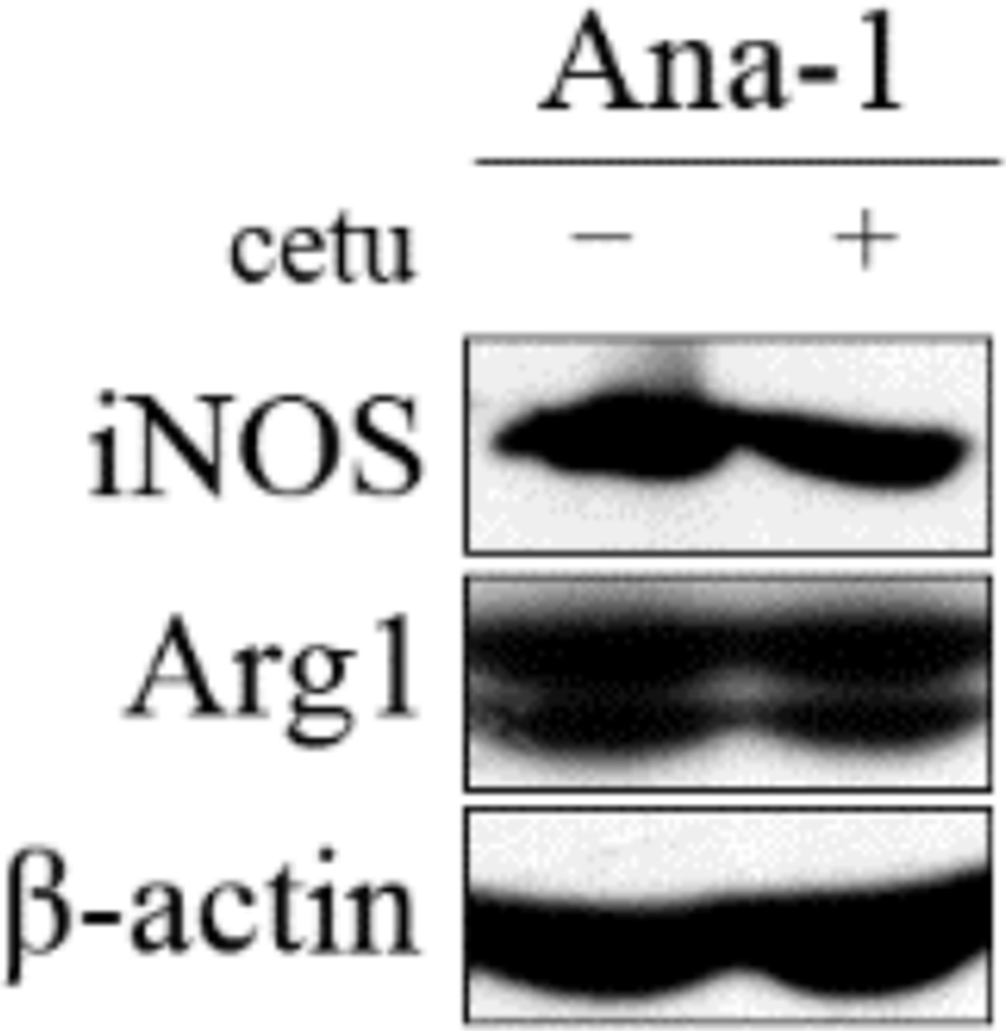

Supplementary Figure S2: Cetuximab had no directly effect on the polarization of Ana-1 cells. Ana-1 cells directly treated cetuximab for 48 h, then Arg1 and iNOS protein levels were detected by Western blot. Cetu: cetuximab, 10 ng/mL.

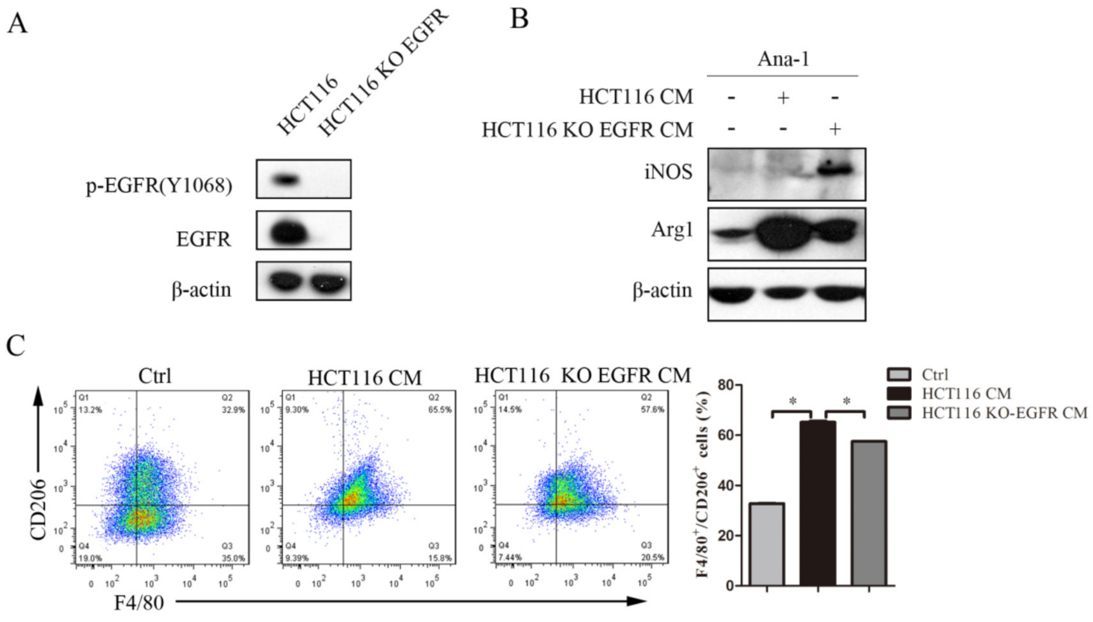

Supplementary Figure S3: HCT116 CM induced Ana-1 cells polarization to M2-like phenotype and was inhibited when EGFR signaling pathway was blocked. A. EGFR protein levels in HCT116 and HCT116 KO-EGFR cells were detected by Western blot. **B.** Arg1 and iNOS protein levels in Ana-1 cells were detected by Western blot after incubation with RPMI1640, HCT116 CM and HCT116 KO-EGFR CM for 48 h. **C.** Percentages of $F4/80^{+}/CD206^{+}$ in Ana-1 cells after incubation with RPMI1640, HCT116 CM and HCT116 KO-EGFR CM for 48h were detected by flow cytometry. Bars represent as mean ± SD (n = 3) for each treatment. $*p < 0.05$; $**p < 0.01$.

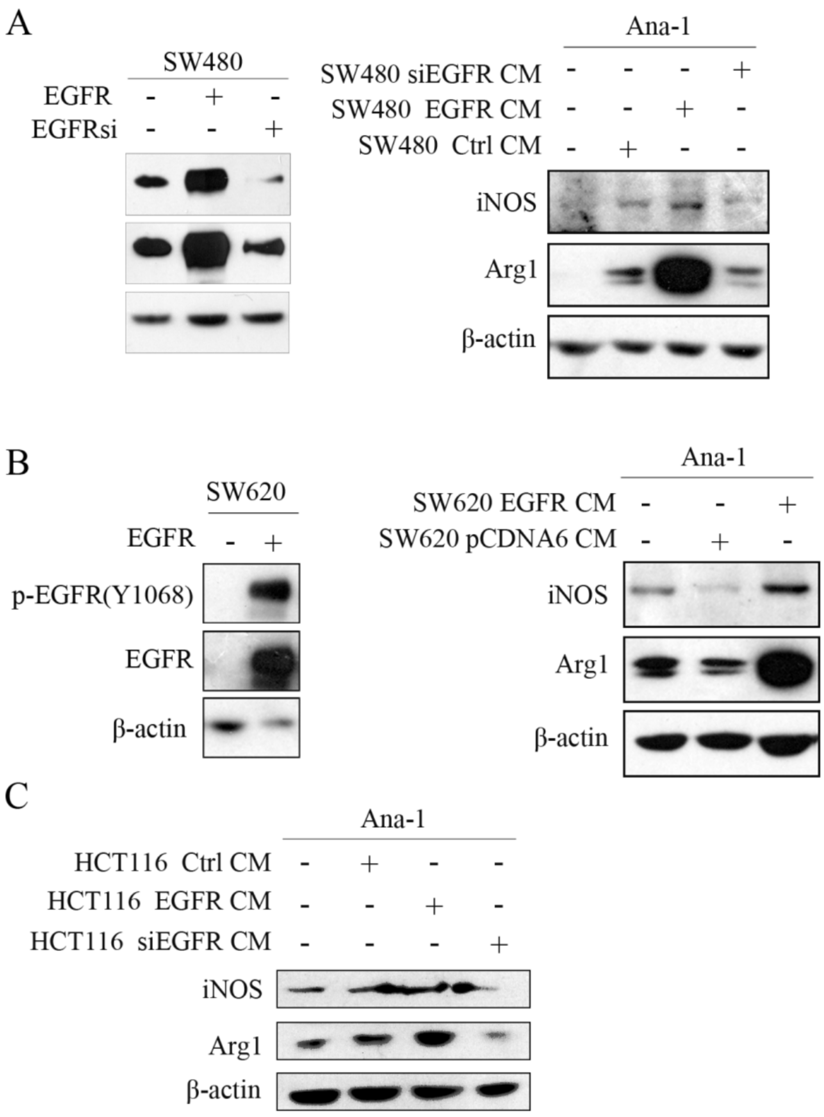

Supplementary Figure S4: Colon cancer CM induced Ana-1 polarization to M2-like phenotype and correlated positively to EGFR signaling pathway. A. EGFR protein levels in SW480, SW480 cells transfected with EGFR siRNA, pCDNA6 EGFR plasmid for 48 h were detected by Western blot. Then, Arg1 and iNOS protein levels in Ana-1 cells were detected by Western blot after incubation with CM from SW480 ctrl, SW480 siEGFR and SW480 EGFR cells for 48 h. **B.** EGFR protein levels in SW620, SW620 cells transfected with pCDNA6 EGFR plasmid were detected by Western blot. Then, Arg1 and iNOS protein levels in Ana-1 cells were detected by Western blot after incubation with CM from SW620, SW620 EGFR cells for 48 h. **C.** Arg1and iNOS protein levels in Ana-1 cells were detected by Western blot after incubation with CM from HCT116 ctrl, HCT116 siEGFR and HCT116 EGFR cells for 48 h.

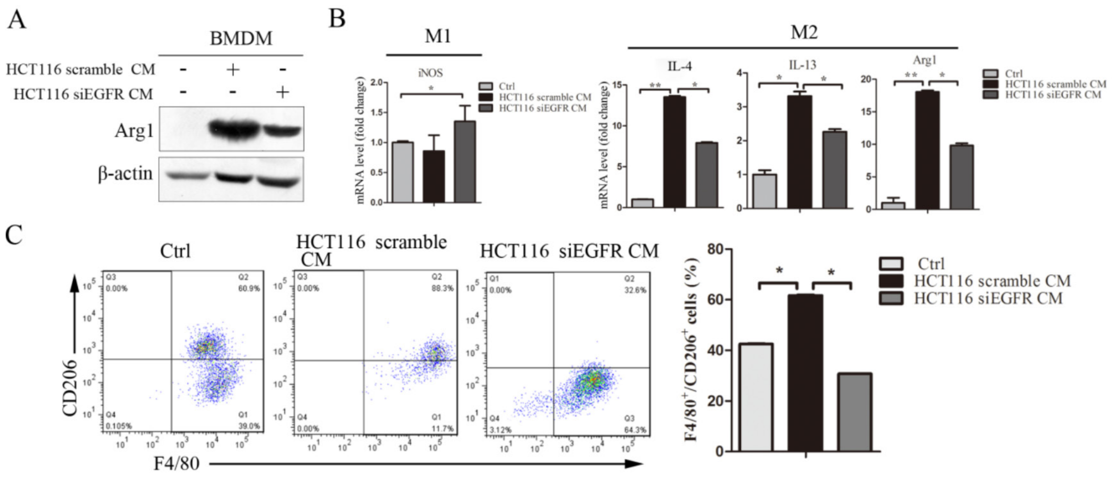

Supplementary Figure S5: HCT116 scramble EGFR CM induced BMDM cells polarization to M2-like phenotype and was inhibited when EGFR signaling pathway was blocked. A. Arg1 protein level in BMDM cells after incubation with CM from HCT116 scramble, HCT116 siEGFR cells was detected by Western blot. **B.** M1 marker iNOS and M2 related markers (IL-4, IL-13 and Arg1) mRNA levels were detected by q-PCR in BMDM cells after incubation with CM from HCT116 scramble, HCT116 siEGFR cells. **C.** Percentages of F4/80$^+$/CD206$^+$ in BMDM cells were detected by flow cytometry after incubation with CM from HCT116 scramble, HCT116 siEGFR cells. Bars represent as mean ± SD (n = 3) for each treatment. *$p < 0.05$; **$p < 0.01$.

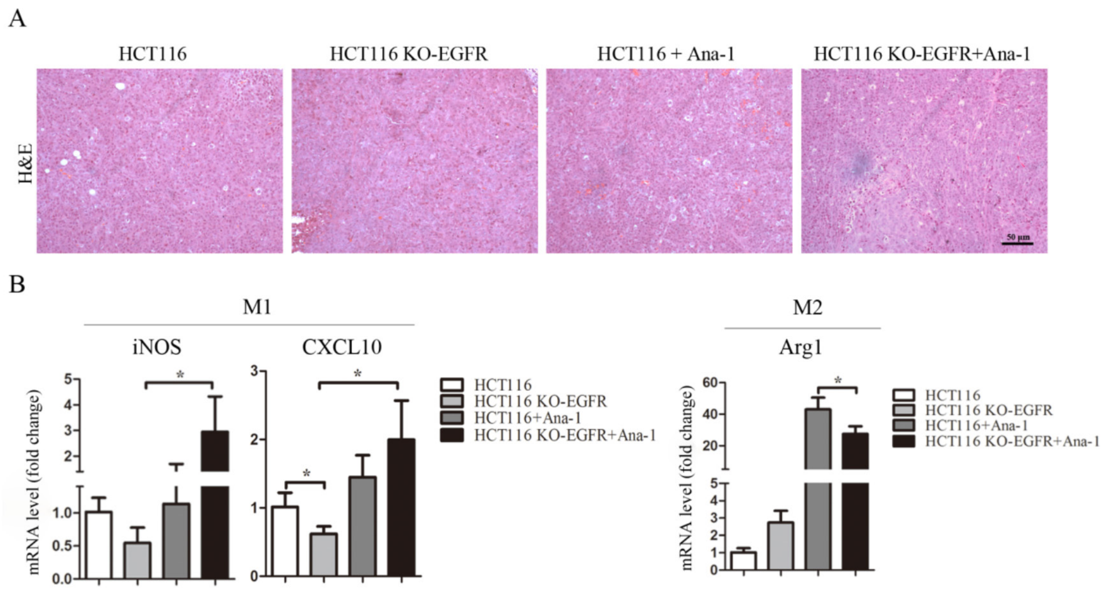

Supplementary Figure S6: HE staining and M1 and M2 related markers mRNA levels detection of xenograft tumor tissues. A. HE staining of xenograft tissues in injected with HCT116, HCT116 KO-EGFR, HCT116 plus Ana-1, HCT116 KO-EGFR plus Ana-1 mice tumor tissues. **B.** M1 related markers (iNOS and CXCL10) and M2 related marker (Arg1) mRNA levels were detected by q-PCR in xenograft tumor tissues. Bars represent as mean ± SD (n = 3) for each treatment. *$p < 0.05$; **$p < 0.01$.

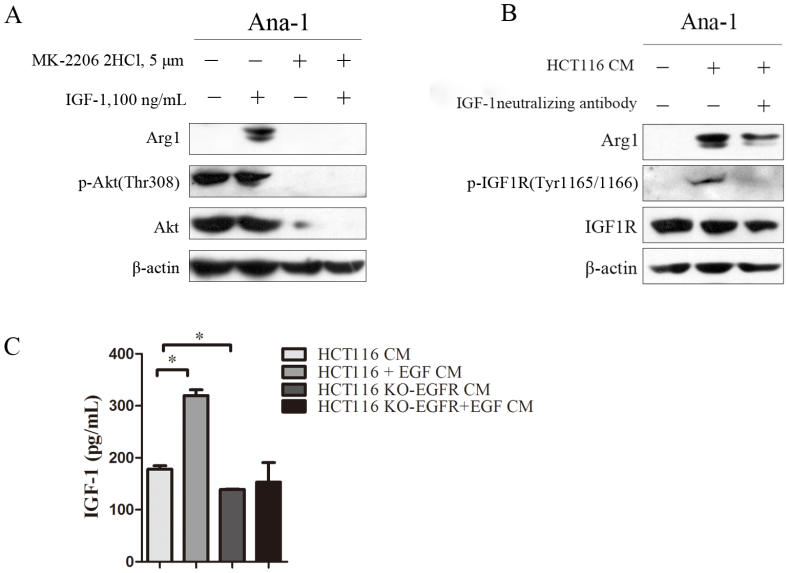

Supplementary Figure S7: Colon cancer-derived IGF-1 contributes to M2-like macrophage polarization. A. Akt inhibitor MK-2206 2HCL reduced the expression of Arg1 induced by IGF-1. Pretreated Ana-1 cells with Akt inhibitor MK-2206 2HCL (5 µm, Selleck Chemicals, Texas, USA) for 30 min, then IGF-1(100 ng/mL) was added to Ana-1 cells for 48 h, the levels of Akt and Arg1 protein were detected by Western blot. **B.** IGF-1 neutralizing antibody decreased the expression of Arg1 induced by HCT116 CM. Pre-mix HCT116 CM with IGF-1 neutralizing antibody (5 µg/mL) for 30 min, then the HCT116 CM was added to Ana-1 cells for 48 h, then the levels of IGF1R and Arg1 protein were detected by Western blot. **C.** IGF-1 concentrations increased in EGF stimulated HCT116 cells. Pretreated HCT1116 cell with EGF for 24h, then fresh medium were replaced and cultured for a further 24 h. IGF-1 concentrations were determined by ELISA. Bars represent as mean ± SD (n = 3) for each treatment. *$p < 0.05$; **$p < 0.01$.
